# Visceral fat area is associated with HbA1c but not dialysate-related glucose load in nondiabetic PD patients

**DOI:** 10.1038/srep12811

**Published:** 2015-08-04

**Authors:** Li-chun Ho, Chung-Jen Yen, Chia-Ter Chao, Chih-Kang Chiang, Jenq-Wen Huang, Kuan-Yu Hung

**Affiliations:** 1 Division of Nephrology, Department of Internal Medicine, I-Shou University, E-DA Road, Kaohsiung, Taiwan; 2 Department of Internal Medicine, National Taiwan University Hospital and College of Medicine, Taipei; 3 Department of Internal Medicine, Jin-Shan branch, National Taiwan University Hospital and College of Medicine, New Taipei City, Taiwan

## Abstract

Factors associated with increased visceral fat area (VFA) have been well documented in the general population but rarely explored in nondiabetic individuals on peritoneal dialysis (PD). As glycosylated hemoglobin (HbA1c) is positively correlated with VFA in diabetic patients, we hypothesized that the same correlation would exist in nondiabetic PD patients. We enrolled 105 nondiabetic patients who had undergone chronic PD for more than 3 months. Each subject underwent an abdominal computed tomography (CT) scan, and the umbilicus cut was analyzed for VFA. VFA values, corrected for body mass index and subjected to natural logarithm transformations, were examined to determine whether they were correlated with HbA1c and other parameters. PD dialysates prescribed at the time of enrollment were recorded to calculate glucose load. We found that when 105 nondiabetic PD patients were classified according to tertiles of HbA1c, higher HbA1c was associated with larger VFA. Multiple linear regression analysis revealed that HbA1c was an independent determinant of VFA, while glucose load and other PD-specific factors were not. In summary, HbA1c, but not PD-related glucose load, was positively correlated with VFA in nondiabetic PD patients, suggesting clinical utility of HbA1c in the PD population.

Robust evidence has suggested a causative role for visceral obesity in insulin resistance, atherogenic dyslipidemia, and adverse cardiovascular events^1^. Visceral adiposity has also been associated with increased cardiovascular risk in hemodialysis (HD) and peritoneal dialysis (PD) patients, and this association remained after adjusting for the confounding effects of diabetes mellitus (DM)^2^,^3^. Etiologies contributing to visceral fat accumulation include old age, male sex, Asian origin, chronic stress, saturated fat intake, and fructose consumption^1^. Antihypertensive medicines may also modulate body fat distribution, as renin-angiotensin system (RAS) blockade reduces visceral fat accumulation, while nonvasodilating beta blockades tend to induce unfavorable cardiometabolic effects^4^,^5^.

The PD population is unique with respect to research involving visceral adiposity because of two characteristics. First, the infusion of glucose-based PD solution results in an extra 100–200 g glucose load per day, which could explain the greater increase in visceral fat area (VFA) observed in PD patients relative to HD patients^6^,^7^. Second, waist circumference may not be a trustworthy indicator of VFA, as the abdomen becomes distended due to accumulation of PD fluid. Although computed tomography (CT) is a reliable means of assessing VFA in PD patients, it is too expensive to be used routinely in clinical settings. A surrogate marker indicating the degree of visceral adiposity present in PD patients is therefore of great use.

Glycosylated hemoglobin (HbA1c) has been shown to be positively correlated with VFA in patients with type 2 DM^8^. Whether this correlation exists in nondiabetic patients is unclear, but several studies have found that elevated HbA1c levels, even those that did not reach the criteria for DM diagnosis, predicted mortality in general nondiabetic populations as well as nondiabetic PD patients^9^,^10^,^11^. Given the detrimental impact of visceral adiposity on PD patients^3^,^12^,^13^, it is quite reasonable to assume that the adverse effects of HbA1c observed in the nondiabetic PD population may be derived from its link to visceral obesity.

The aim of this study was to examine the association between VFA and HbA1c in nondiabetic PD patients. We intended to prove the association by showing a dose-dependent effect of VFA in patients grouped according to HbA1c tertile, and a statistically significant result in a correlation test for HbA1c and VFA. Furthermore, the independency of HbA1c as a determinant for VFA was tested in a linear model adjusting for the clinical parameters related to visceral adiposity.

## Methods

### Study design

Nondiabetic dialysis patients who were older than 20 years of age and had undergone chronic PD for more than 3 months were enrolled in the study in 2009. The definition of nondiabetic status in this study was HbA1c ≤ 6.4% without a history of anti-diabetic treatment. Pregnant women and patients who had undergone CT scan during the preceding 6 months were excluded from study participation. After providing informed consent, each patient underwent an abdominal CT scan and blood sample collection. Patients refrained from eating for 6 h prior to undergoing phlebotomy. The blood samples were immediately centrifuged at 3,000 rpm and 4 °C. The plasma samples were frozen at −80 °C until analysis.

### Ethical considerations

This study was approved by the ethics committee of National Taiwan University hospital in NTUH-REC No. 200808062R and NTUH-REC No. 201104032RC. The study was carried out in accordance with the approved guidelines. Patients provided written informed consent prior to participating in the study.

### Assessment of abdominal fat using computed tomography

Imaging was performed using a 64-MDCT scanner (LightSpeed VCT; GE Healthcare, Milwaukee, WI), and the umbilicus cut was analyzed for VFA^14^. Image analysis software (ImageJ, version 1.45; National Institutes of Health, Bethesda, MD) was used at an attenuation range of −50 to −250 Hounsfield units to quantify abdominal adipose tissue areas in cm^2^. Subcutaneous fat area (SFA) was clearly visible and defined as extraperitoneal fat between skin and muscle. Intra-abdominal tissue at the same density as SFA was defined as VFA. The sum of SFA and VFA represented the total fat area (TFA). The images were reviewed by radiologists who were unaware of the clinical characteristics of the PD patients. These indicators for fat area were all corrected for BMI (units, cm^2^/[kg/m^2^]), because fat area is related to body size^15^.

### HbA1c and other clinical characteristics

Blood HbA1c level of each PD patient was measured by Boronate affinity chromatography as described in our previous report^16^. Other clinical data were obtained via anthropometric measurement, biochemical study for the plasma samples, and reviewing the medical records for PD treatment. These clinical characteristics were classified into 5 types: (1) Demographic and anthropometric factors related with visceral adiposity, including age, sex, BMI, and lean body mass estimated by creatinine kinetics^1^,^17^. (2) Factors associated with glucose metabolism and visceral fat accumulation, including fasting plasma glucose, plasma insulin level, and insulin resistance represented by homeostasis model assessment (HOMA_IR_)^8^,^18^,^19^. (3) PD-specific factors included PD duration, peritoneal urea clearance (Kt/V), residual renal function, and the results of peritoneal equilibration test. In addition, records detailing the dialysate that was prescribed to each PD patient in 2009 were used to calculate glucose load according to a method described previously^20^,^21^,^22^. Glucose load 1 represented the average glucose concentration in total PD fluid including Extraneal and Nutrineal. Glucose load 2 represented the average glucose concentration of glucose-based dialysate alone. (4) Patients who had received RAS blockades or beta blockades for more than 3 months at the time of enrolment were recorded as RAS or beta blockade users. (5) Cardiovascular risk-related factors in PD patients included nutrition markers (i.e. albumin, normalized protein catabolic rates [nPCR], and creatinine), inflammatory markers (i.e. C-reactive protein [CRP]), lipid metabolic factors (i.e. cholesterol [CHO], triglyceride [TG], low density lipoprotein [LDL], and high density lipoprotein [HDL]), and atherogenic indices (AIs)^23^,^24^. Two AIs were derived from the lipid profile according to the following equations: AI1 = log (TG/HDL-C)^25^ and AI2 = non-HDL cholesterol/HDL = (T-CHO-HDL)/HDL^15^.

### Statistical analysis

All continuous variables were reported as means ± SDs (with 95% confidence intervals as appropriate), and all categorical variables were reported as frequencies or percentages. Comparison between two groups was performed using the two-sided Student t test whereas comparison among three or more groups (e.g. groups according to HbA1c tertile) was using one way analysis of variance (ANOVA). The differences in sex distribution were tested using χ^2^ analysis. The relationships between variables were assessed using Pearson’s correlation coefficients. The independent determinants of the variables were examined via multiple linear regression analysis. Variables were selected via a stepwise procedure with backward selection. A p value of 0.1 was used as the limit for entry into the model. For all statistical testing, p values of  <0.05 were considered significant unless otherwise specified. Statistical analyses were conducted using SPSS 13.0 for Windows (SPSS Inc., IL, USA).

## Results

### Comparing VFA among groups classified according to HbA1c tertiles

**Figure 1:**
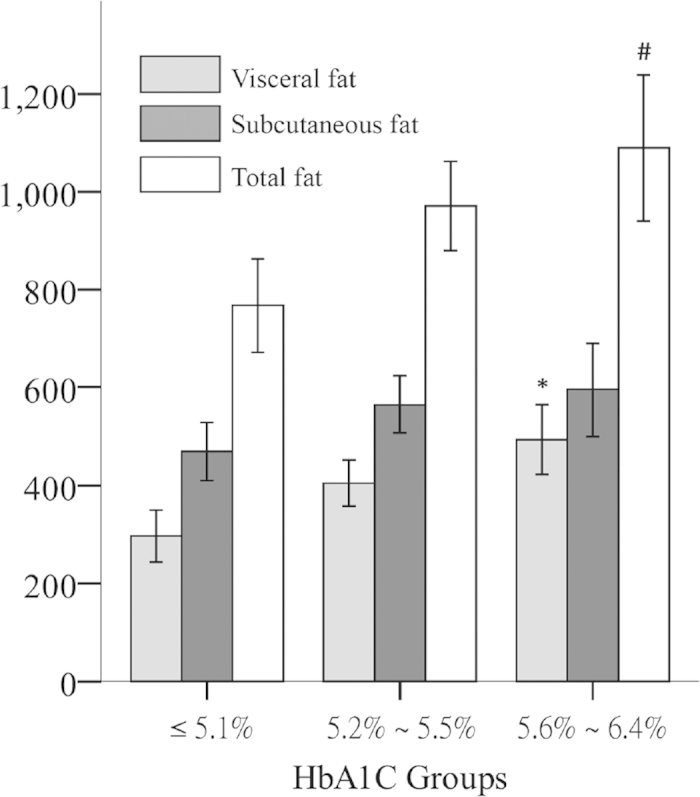
Comparison of fat tissue areas between the three PD groups classified according to HbA1C tertiles. Comparisons among the three groups were performed using ANOVA. *Significant difference (p ≤ 0.05) in visceral fat area between the three groups; ^#^Significant difference (p ≤ 0.05) in total fat area between the three groups.

A total of 138 nondiabetic PD patients who met the study criteria underwent abdominal CT. Of these patients, 105 underwent blood sample collection and analysis. The patients were classified into 3 groups according to HbA1C tertiles: Group 1: HbA1c ≤ 5.1% (N = 36), Group 2: HbA1c = 5.2–5.5% (N = 34), and Group 3: HbA1c = 5.6–6.4% (N = 35). Higher HbA1c was associated with larger TFA (P = 0.013) and VFA (P = 0.002) in nondiabetic PD patients, while the differences in SFA between groups did not reach statistical significance (P = 0.17) (Fig. 1). Besides the increased in VFA, patients with a higher HbA1c level also tended to be older (P = 0.052) and demonstrated a trend toward impaired glucose metabolism (higher fasting glucose [P = 0.001] and HOMA_IR_ [P = 0.001]) (Table 1). In contrast, PD-specific factors, including PD duration and PD glucose load, were unrelated to HbA1c tertile (Table 1). The only exception was peritoneal urea clearance (peritoneal Kt/V), which reversely correlated with HbA1c tertile (P = 0.048). Only two cardiovascular risk-related factors, creatinine (P = 0.043) and CRP (P = 0.041), were associated with HbA1c tertile. Interestingly, it was the group with the middle HbA1c tertile had the highest serum creatinine and the lowest CRP level, suggesting more muscle mass and less inflammatory status in this group than in the other two groups (Table 1).

### Correlations between HbA1c, VFA and other clinical parameters

The associations between HbA1c and VFA and other variables were analyzed using Pearson correlation coefficients. As VFA and CRP were not normally distributed, we used the natural logarithm transformations of two variables. Table 2 showed that HbA1c and lnVFA were positively correlated with each other (p < 0.001) and age (p = 0.01 for HbA1c, p < 0.001 for lnVFA), BMI (p = 0.02 for HbA1c, p < 0.001 for lnVFA), glucose metabolic factors like HOMA_IR_ (p < 0.001 for both HbA1c and lnVFA) and serum insulin level (p < 0.001 for both HbA1c and lnVFA), and negatively correlated with percentage of lean body mass (p = 0.01 for HbA1c, p < 0.001 for lnVFA). PD glucose load and other PD-specific factors were not significantly correlated with HbA1c or lnVFA except that HbA1c was negatively correlated with peritoneal Kt/V (p = 0.02). As for cardiovascular risk-related factors, both HbA1c and lnVFA were positively correlated with lnCRP (p = 0.01 for HbA1c, p < 0.001 for lnVFA) and AI1 (p = 0.03 for HbA1c, p < 0.001 for lnVFA), whereas only lnVFA but not HbA1c was positively correlated with nutritional markers albumin (p = 0.02), nPCR (p = 0.03), and atherogenic marker AI2 (p < 0.001).

### The effects of RAS and beta blockades on HbA1c and VFA

**Figure 2:**
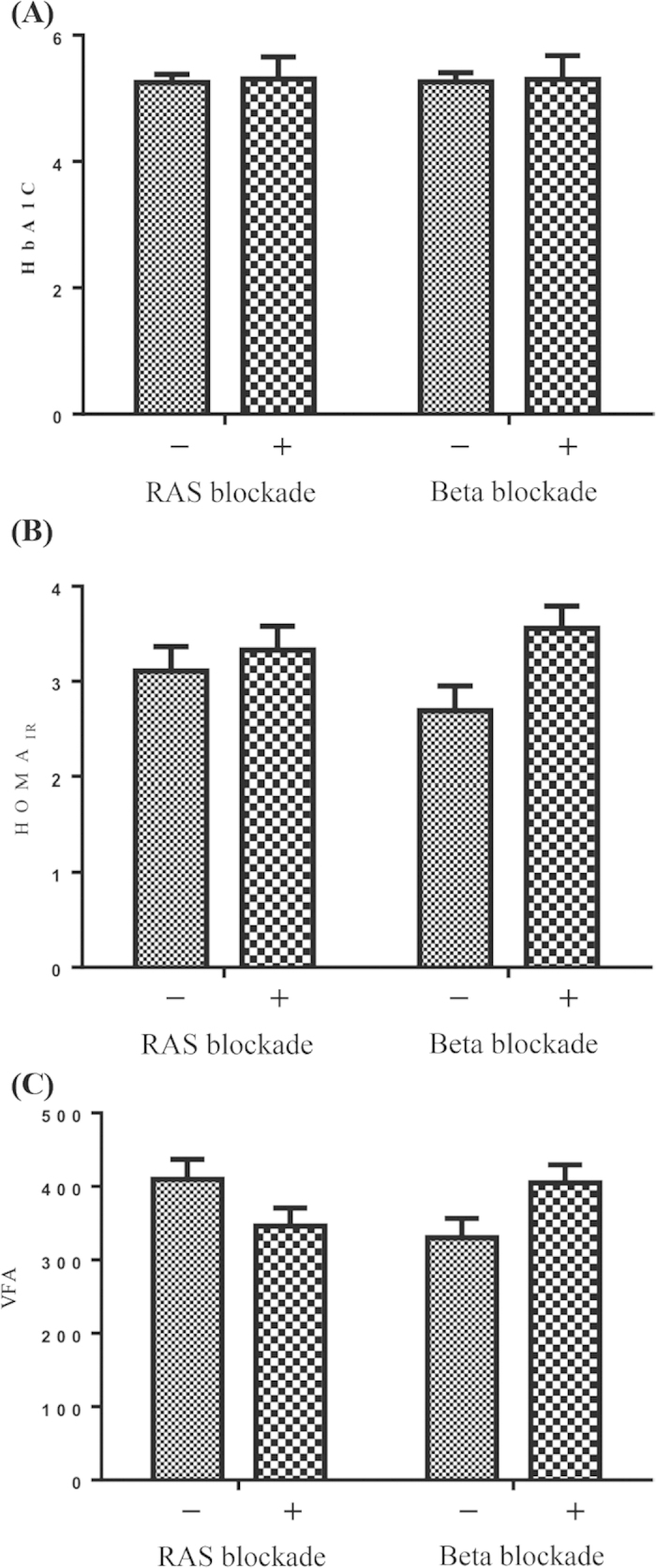
The effects of RAS and beta blockades on insulin resistance and visceral fat. Patients who did not take RAS blockades: n = 50, patients taking RAS blockades: n = 55; patients who did not take beta blockades: n = 45; patients taking beta blockades: n = 60. Comparisons between the users and nonusers were performed using Student t test. HbA1c: glycosylated hemoglobin; HOMA_IR_: homeostasis model assessment-insulin resistance; VFA: visceral fat area.

RAS and beta blockades may affect glucose and lipid metabolism and consequently alter HbA1c and VFA^4^,^5^. Figure 2 shows that RAS- and beta-blockade users displayed similar HbA1c levels and HOMA_IR_ to nonusers (RAS-blockade users vs. nonusers, HbA1c: 5.3 ± 0.4% vs. 5.3 ± 0.5%, p = 0.52, HOMA_IR_: 3.33 ± 1.88 vs. 3.11 ± 1.80, p = 0.55; beta-blockade users vs. nonusers, HbA1c: 5.3 ± 0.4% vs. 5.3 ± 0.5%, p = 0.65, HOMA_IR_: 3.56 ± 1.88 vs. 2.69 ± 1.65, p = 0.17). However, RAS and beta blockades appeared to exert an impact on VFA in our patients. Patients taking RAS blockades showed a trend toward lower VFA relative to those who did not take RAS blockades (346 ± 184 mm^2^/kg/m^2^ vs. 410 ± 192 mm^2^/kg/m^2^, p = 0.09), while the patients taking beta blockades exhibited significantly higher VFA relative to those who did not take beta blockades (405 ± 199 mm^2^/kg/m^2^ vs. 330 ± 167 mm^2^/kg/m^2^, p = 0.05).

### Independent determinants of VFA

The independent determinants of lnVFA were analyzed with multiple linear regression. All available variables were introduced into the regression model and were selected by a stepwise procedure. Table 3 listed only the variables with sufficient significance to remain in the model. HbA1c was positively and independently associated with lnVFA (p = 0.005). BMI remained an independent predictor of lnVFA (p = 0.002), even though VFA had been corrected for BMI. Age (p = 0.045) and AI1 (p = 0.002) were positively associated with lnVFA while RAS blockade use (p = 0.046) and serum creatinine levels (p = 0.022) were negatively associated with lnVFA in nondiabetic PD patients (R^2^ = 0.484, Table 3).

## Discussion

This cross-sectional study aimed to reveal the determinants of VFA in a nondiabetic PD population. Our results showed a positive and independent association between HbA1c and VFA, while PD-specific factors, glucose load, and peritoneal Kt/V were not significantly correlated with VFA.

Linking HbA1c to VFA may expand the clinical utility of HbA1c for PD patients. A positive correlation between HbA1c and VFA has been shown in nondiabetic, nondiaysis populations^19^,^26^,^27^, and the present study found the same correlation in nondiabetic PD patients. Given the harmful effects of visceral fat accumulation^1^, the link between HbA1c and VFA may explain the predictive power of HbA1c with respect to mortality and adverse outcomes in general nondiabetic populations^9^,^10^,^28^ as well as nondiabetic PD patients^11^,^29^. Our results were not only compatible with our previous finding that body composition affects PD patients’ survival^30^ but also suggested that, in nondiabetic PD patients, HbA1c is a potential surrogate marker for VFA, prompting physicians to initiate early action against visceral obesity.

Among factors associated with VFA, several were also correlated with HbA1c (Table 2). Old age and high BMI have been acknowledged as etiologies of visceral fat accumulation^1^, and the adverse influence of visceral adiposity on glucose and lipid metabolism could contribute to positive correlations between VFA and insulin resistance (HOMA_IR_), HbA1c, and atherogenic risk (AI). Although lower VFA and HbA1c generally mean less obesity and healthier nutritional status, it is interesting that the highest creatinine (indicating muscle mass) and lowest CRP were not observed in patients with the lowest VFA and HbA1c but were present in those with HbA1c in the second tertile (Table 1). This result was compatible with findings from previous studies in which HbA1c of less than 5.1% was associated with increased mortality in a general nondiabetic population and nondiabetic PD patients^9^,^11^. The J-shape association curve is generally referred to as protein-energy wasting, a phenomenon in which malnutrition is associated with inflammation and survival disadvantages^31^. Nevertheless, visceral adiposity is still an important therapeutic target, and some studies have suggested the use of RAS blockades or avoidance of beta blockades to decrease visceral fat accumulation^4^,^5^. The benefits of RAS blockades and disadvantages of beta blockades with respect to VFA were also suggested in our study subjects (Fig. 2, Table 3), although a clear cause-effect relationship could not be confirmed in a cross-sectional study like the present one. Despite the above-mentioned complex associations between HbA1c and multiple risk factors, HbA1c remained independently associated with VFA in the multiple linear regression model (Table 3), implying a fundamental role for VFA in inducing insulin resistance in nondiabetic PD patients.

The current study did not find an association between VFA and PD-related glucose load. In our previous study, involving both diabetic and nondiabetic PD patients, high glucose load was associated with poor survival^20^. As PD patients showed greater longitudinal increase in visceral fat and poorer glucose metabolism relative to HD patients^7^,^32^, our preliminary hypothesis was that glucose load related to PD fluid would affect survival by inducing visceral fat accumulation and aggravating insulin resistance. However, our data showed that neither glucose load nor PD duration was associated with VFA or HbA1c (Tables 1 and 2). PD transport characteristics, which constitute a variable that is highly correlated with glucose load, were also analyzed in the linear regression analysis but were not relevant with respect to VFA (data not shown). It is probable that, in nondiabetic patients, glucose load did not exceed the metabolic capacity to handle it; therefore, it did not have a marked effect on visceral adiposity.

There were some limitations to this study. It was cross-sectional in design; therefore, prospective changes in VFA and HbA1c were unknown. All the participants were Asian origin, which has been acknowledged as a risk factor for visceral fat accumulation; thus the generalizability of our conclusions may be limited. Saturated fat intake and fructose consumption were not recorded for our subjects; however, the metabolic consequences of these nutrients (atherogenic lipid profile and insulin resistance) were well analyzed in our study. Several conditions present in PD patients may disturb the accuracy of HbA1c (e.g., iron deficiency, vitamin B12 deficiency, and uremia)^33^. Although we did not adjust for these disturbing factors, a previous study identified HbA1c as a reliable index of glycemic control in diabetic PD patients^16^; therefore, the reliability of HbA1c in nondiabetic PD patients may be acceptable.

In summary, our study showed that HbA1c and VFA were independently and positively associated with each other in nondiabetic PD patients. These results suggested clinical utility of HbA1c as a surrogate marker for VFA in nondiabetic PD patients and highlighted the need for further research to study the prognostic value of HbA1c in PD populations.

## Additional Information

**How to cite this article**: Ho, L.-c. *et al*. Visceral fat area is associated with HbA1c but not dialysate-related glucose load in nondiabetic PD patients. *Sci. Rep.* **5**, 12811; doi: 10.1038/srep12811 (2015).

## Figures and Tables

**Table 1:** Clinical characteristics of the three peritoneal dialysis groups classified according to HbA1C tertiles.

	Glycosylated hemoglobin category		
	≤5.1%	5.2–5.5%	5.6–6.4%
TFA (mm^2^/Kg/m^2^)*	794 ± 312	913 ± 372	1052 ± 399
SFA (mm^2^/Kg/m^2^)	497 ± 205	532 ± 223	598 ± 260
VFA (mm^2^/Kg/m^2^)*	297 ± 172	380 ± 183	454 ± 186
Sex (men/women)	10/26	15/19	14/21
Age (years)*	48 ± 16	52 ± 13	54 ± 10
BMI (kg/m^2^)	21.7 ± 2.8	22.9 ± 3.2	23.1 ± 3.0
Glucose (mg/dL)*	94 ± 15	94 ± 16	108 ± 27
HOMA_IR_*	2.70 ± 1.75	2.82 ± 1.25	4.17 ± 2.06
PD Vintage (months)	55 ± 45	48 ± 46	47 ± 40
Peritoneal Kt/V*	2.05 ± 0.46	1.92 ± 0.34	1.83 ± 0.25
Renal Kt/V	0.20 ± 0.30	0.16 ± 0.28	0.18 ± 0.24
Glucose load 1(g/dL)	1.67 ± 0.27	1.69 ± 0.26	1.66 ± 0.32
Glucose load 2 (g/dL)	1.85 ± 0.33	1.84 ± 0.31	1.86 ± 0.32
nPCR (g/Kg/day)	1.02 ± 0.16	0.93 ± 0.16	0.96 ± 0.22
Albumin (g/dL)	4.0 ± 0.4	4.1 ± 0.4	4.1 ± 0.3
Creatinine (mg/dL)*	10.9 ± 2.6	12.3 ± 3.1	11.0 ± 2.7
CRP (mg/dL)*	0.84 ± 1.38	0.41 ± 0.57	1.69 ± 3.35
Cholesterol (mg/dL)	202 ± 56	194 ± 39	205 ± 55
Triglyceride (mg/dL)	170 ± 101	179 ± 157	245 ± 253
AI1	0.52 ± 0.35	0.53 ± 0.37	0.66 ± 0.34
AI2	3.76 ± 1.36	3.83 ± 1.38	4.01 ± 1.62

Differences among the 3 groups were analyzed using ANOVA, except that sex was analyzed using χ^2^ test. *P ≤ 0.05.

TFA: total fat area; SFA: subcutaneous fat area; VFA: visceral fat area; BMI: body mass index; HOMA_IR_: homeostasis model assessment-insulin resistance; PD: peritoneal dialysis; Kt/V: urea clearance; nPCR: normalized protein catabolic rate; CRP: C-reactive protein; AI1: atherogenic index 1; AI2: atherogenic index 2.

**Table 2:** Correlations between HbA1c and VFA and other clinical parameters.

	HbA1c	p	lnVFA	p
lnVFA	0.41	<0.001	—	
HOMA_IR_	0.35	<0.001	0.29	<0.001
Age	0.26	0.01	0.45	<0.001
BMI	0.22	0.02	0.33	<0.001
LBM%	−0.26	0.01	−0.42	<0.001
Insulin	0.31	<0.001	0.31	<0.001
PD duration	−0.07	0.47	0.04	0.69
Peritoneal Kt/V	−0.23	0.02	−0.09	0.38
Renal Kt/V	−0.06	0.55	−0.03	0.80
Glucose load 1	0.01	0.94	0.03	0.75
Glucose load 2	0.09	0.34	0.07	0.49
Albumin	0.17	0.08	0.24	0.02
nPCR	−0.17	0.09	−0.22	0.03
lnCRP	0.24	0.01	0.37	<0.001
AI1	0.21	0.03	0.43	<0.001
AI2	0.17	0.07	0.35	<0.001

The correlation was assessed using Pearson’s correlation coefficients.

VFA: visceral fat area; HOMA_IR_: homeostasis model assessment-insulin resistance; BMI: body mass index; LBM: lean body mass; PD: peritoneal dialysis; Kt/V: urea clearance; nPCR: normalized protein catabolic rate; CRP: C-reactive protein; AI1: atherogenic index 1; AI2: atherogenic index 2.

**Table 3:** Independent determinants of visceral fat in nondiabetic peritoneal dialysis patients analyzed via multiple linear regression.

	B ± SE	95%CI	P
HbA1c	0.330 ± 0.114	0.103–0.557	0.005
BMI	0.050 ± 0.016	0.019–0.082	0.002
Age	0.008 ± 0.004	0.000–0.016	0.045
LnCRP	0.052 ± 0.031	−0.010–0.114	0.096
AI1	0.428 ± 0.131	0.167–0.689	0.002
RAS blockade	−0.184 ± 0.091	−0.364–−0.003	0.046
Creatinine	−0.040 ± 0.017	−0.075–−0.006	0.022
Constant	2.738 ± 0.630	1.488–3.989	<0.001

R^2^ = 0.484.

AI: atherogenic index, BMI: body mass index, CRP: C-reactive protein, RAS: rennin-angiotensin system.
